# Analysis of content, quality, and reliability of melanoma-related Chinese videos on TikTok: A cross-sectional study

**DOI:** 10.1097/MD.0000000000046045

**Published:** 2025-11-28

**Authors:** Landong Ren, Qinxiao Li, Miao Qin, Kaidi Zhao

**Affiliations:** a Department of Dermatology, Xi’an No.9 Hospital, Xi’an, China; b Department of Dermatology, Xi’an Jiaotong University Second Affiliated Hospital, Xi’an, China.

**Keywords:** health communication, information quality, melanoma, social media, tiktok

## Abstract

Melanoma is the most aggressive form of skin cancer, with a rising incidence worldwide. Social media platforms such as TikTok are increasingly serving as important channels for health information dissemination, yet the quality and reliability of melanoma-related content remain unclear. This study evaluated the characteristics, content distribution, quality, and reliability of Chinese-language melanoma-related short videos on TikTok. From August 7 to 10, 2025, 113 melanoma-related videos were collected. Video characteristics were recorded, and the global quality score and modified DISCERN (mDISCERN) tool were used for evaluation. The videos were generally short (median: 58.00 seconds) and had high engagement. Most content focused on risk factors, clinical manifestations, and treatment, with limited coverage of prevention and recurrence. Overall quality and reliability were low, with median global quality score and mDISCERN scores of 2.00 (interquartile range: 2.00–2.00). Significant differences in quality were observed among uploader types, with dermatologists producing the highest-quality content. No significant correlation was found between engagement metrics and quality. The quality and reliability of melanoma-related videos on TikTok are suboptimal. This study provides empirical evidence on the current status of melanoma health information in the social media environment and offers a reference for optimizing digital health communication strategies. Future efforts should enhance the comprehensiveness and scientific rigor of health content, increase the participation of healthcare professionals, and establish platform-level quality control mechanisms to ensure the accuracy and reliability of health information.

## 1. Introduction

Melanoma is a highly malignant tumor originating from melanocytes and represents the most aggressive form of skin cancer, accounting for 65% of skin cancer–related deaths, with poor prognosis at advanced stages.^[1,2]^ The incidence of melanoma has continued to rise in recent years, making it a substantial public health concern worldwide.^[3,4]^ Given that the disease has a high metastatic potential, once diagnosed at an advanced stage, the prognosis becomes dismal, with a markedly shortened median survival time.^[5,6]^ Early detection and timely treatment are therefore essential for improving patient survival rates and reducing the overall disease burden.^[7–9]^ Raising public awareness of melanoma’s risk factors, early clinical manifestations, and preventive measures can facilitate early intervention, standardized treatment, and improved prognosis, while also alleviating the associated social and economic burden.^[10–12]^ Consequently, the efficient dissemination of accurate and reliable health information to the public has become a critical component of melanoma prevention and control strategies.

With the rapid development of digital media, social media platforms have become one of the primary channels for the public to access health-related information.^[13–15]^ TikTok is one of the most popular short-video platforms worldwide, characterized by its massive user base, algorithm-driven personalized recommendations, and high interactivity.^[16,17]^ These features enable rapid and extensive dissemination of medical information but also raise concerns about the accuracy, reliability, and balance of the content. Previous studies have indicated that short videos on TikTok related to orthognathic surgery,^[18]^ epidural blood patch,^[19]^ cataracts,^[20]^ and nonalcoholic fatty liver disease^[21]^ often achieve high engagement but generally exhibit suboptimal quality and reliability. Such content may contribute to the spread of misinformation, potentially delaying accurate diagnosis and appropriate treatment.

Given the rising incidence of melanoma in China and the increasing reliance on social media for health information, it is necessary to evaluate the current status of melanoma-related short videos on TikTok. This study aims to analyze the content distribution, quality, and reliability of these videos, compare differences among uploader types, and explore the associations between audience engagement metrics and information quality. The findings are expected to provide evidence-based insights to optimize digital health communication strategies and inform public health interventions on social media platforms.

## 2. Methods

### 2.1. Ethics approval

This study does not involve human subjects, clinical data, laboratory animals, or histological research. All the data analyzed were obtained from publicly available TikTok videos, and data collection was conducted in strict accordance with TikTok’s terms of service. No private or personally identifiable information was collected or processed, and no interaction with users was conducted. Therefore, ethical approval was not required.

### 2.2. Study design

This cross-sectional study was conducted from August 1 to August 4, 2025, aiming to evaluate the content, quality, and reliability of short videos related to melanoma on the TikTok platform.

### 2.3. Data source and search strategy

This study used TikTok (Douyin in mainland China) as the data source. The search was conducted on August 7 using the Chinese keyword “黑素瘤” (“melanoma”), and general data on video characteristics, engagement metrics, and uploader identity were collected. To minimize bias introduced by TikTok’s personalized recommendation algorithm, all searches were conducted using a newly registered account on TikTok, with cleared browser cache and no prior browsing history. Only 1 new account was created, as data collection for the top 150 default-ranked videos was completed within 1 day, ensuring that the results were not affected by fluctuations in platform algorithms or browsing history.

### 2.4. Inclusion and exclusion criteria

The videos included in the study must meet the following criteria. First, the videos must be in Chinese. Second, the videos must contain content related to melanoma. On the other hand, videos were excluded if they met any of the following criteria. First, duplicate videos were excluded. Second, videos whose main content was unrelated to melanoma (e.g., those focused on other dermatologic or oncologic conditions) were also excluded.

### 2.5. Data extraction

**Figure 1.**
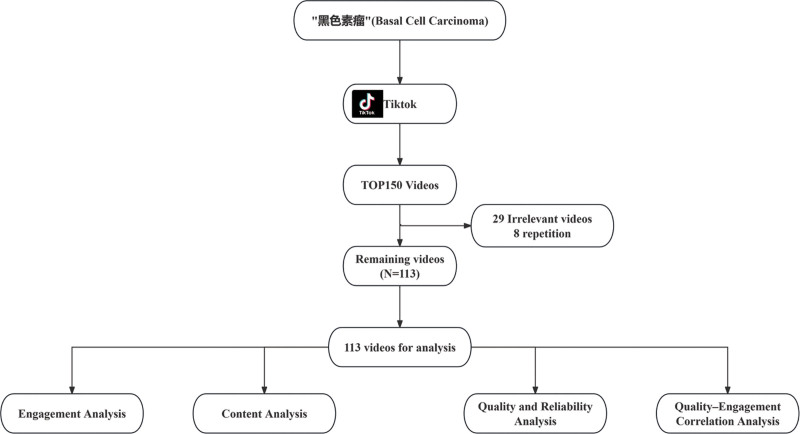
Flow diagram illustrating the selection process and analytical framework for melanoma-related videos on TikTok.

For each included video, the following characteristics were extracted: video duration (in seconds), number of likes, number of favorites, number of comments, number of shares, and uploader type. These characteristics were collected to assess the popularity and interactivity of the videos. The number of likes, favorites, comments, and shares provides insight into how well the video resonates with the audience, while the uploader type helps assess the characteristics of videos uploaded by different individuals. Additionally, thematic content categories were identified, including risk factors, clinical presentation, diagnosis, treatment, prevention, prognosis, and recurrence. These categories were selected to ensure that all relevant aspects of melanoma were addressed in the videos, allowing us to evaluate the comprehensiveness and educational value of the content. The flow diagram of this study is shown in Figure 1.

### 2.6. Quality and reliability assessment

The overall quality of each video was assessed using the global quality scale (GQS),^[22]^ a 5-point Likert scale evaluating structure, ease of use, and comprehensiveness. Reliability was assessed using the modified DISCERN (mDISCERN) instrument,^[23]^ which evaluates clarity of aims, citation of information sources, objectivity of content, and provision of additional information. Both tools have been widely applied in studies of health-related social media content. Detailed scoring criteria for these instruments are provided in Tables 1 and 2. Each video was independently evaluated by 2 trained raters, and any disagreements were resolved by a third rater. The GQS and mDISCERN ratings for melanoma-related videos were mainly completed between August 8 and August 10.

**Table 1:** The GQS quality criteria.

Item features	Points
Poor quality; poor flow of the videos; most information missing; not at all useful for patients	1
Generally poor quality; some information listed, but many important topics missing; of very limited use to patients	2
Moderate quality; suboptimal flow; some important adequately discussed, but other information poorly discussed; somewhat useful for patients	3
Good quality and generally good flow; most of the relevant information listed, but some topics not covered; useful for patients	4
Excellent quality and flow; very useful for patients	5

GQS = global quality score.

**Table 2:** The mDISCERN quality criteria.

Reliability score
1. Is the video clear, concise, and understandable?
2. Are valid sources cited?
3. Is the content presented balanced and unbiased?
4. Are additional sources of content listed for patient reference?
5. Are areas of uncertainty mentioned?

mDISCERN = modified DISCERN.

### 2.7. Statistical analysis

Descriptive statistics were used to summarize video characteristics. Continuous variables with a non-normal distribution were expressed as the median and interquartile range (IQR), while categorical variables were presented as frequencies and percentages. The Kruskal–Wallis test was used to compare parameters among different uploader groups. Spearman rank correlation coefficient was applied to assess associations between engagement metrics and quality/reliability scores. Statistical analyses were performed using R software (version 4.3.2). A 2-tailed *P* value <.05 was considered statistically significant.

## 3. Results

### 3.1. General characteristics of melanoma-related videos

A total of 113 short videos related to melanoma were included in this study. The videos were relatively short in duration (median: 58.00 seconds, IQR: 41.00–82.00). Engagement metrics were relatively high, with the number of likes (median: 566.00, IQR: 208.00–2596.00), favorites (median: 167.00, IQR: 55.00–715.00), comments (median: 146.00, IQR: 28.00–729.00), and shares (median: 132.00, IQR: 35.00–776.00). The overall quality and reliability of the videos were suboptimal, with a median GQS score of 2.00 (IQR: 2.00–2.00) and a median mDISCERN score of 2.00 (IQR: 2.00–2.00). Detailed parameters are presented in Table 3.

**Table 3:** General information, quality, and reliability scores of melanoma-related videos on TikTok.

Variables	TikTok (N = 113)
General information	
Video length(s), M (Q_1_, Q_3_)	58.00 (41.00, 82.00)
Likes, M (Q_1_, Q_3_)	566.00 (208.00, 2596.00)
Collections, M (Q_1_, Q_3_)	167.00 (55.00, 715.00)
Comments, M (Q_1_, Q_3_)	146.00 (28.00, 729.00)
Shares, M (Q_1_, Q_3_)	132.00 (35.00, 776.00)
Video content (n) (%)	
Precipitating factors	55 (48.67%)
Clinical presentation	67 (59.29%)
Diagnosis	25 (22.12%)
Treatment	48 (42.48%)
Prevention	5 (4.42%)
Prognosis	29 (25.66%)
Recurrence	5 (4.42%)
Video quality	
GQS score, M (Q_1_, Q_3_)	2.00 (2.00, 2.00)
mDISCERN score, M (Q_1_, Q_3_)	2.00 (2.00, 2.00)

### 3.2. Content distribution of melanoma-related short videos

**Figure 2.**
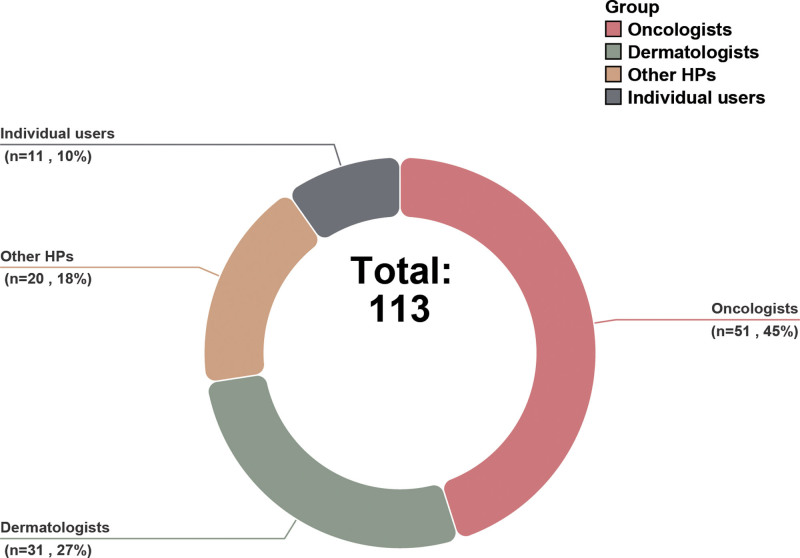
Proportional distribution of uploader categories for included videos.

The most frequently covered topics in melanoma-related short videos were risk factors, clinical presentation, and treatment, whereas prevention and recurrence were the least frequently mentioned (Table 3). The detailed distribution of these content categories is shown in Figure 2.

### 3.3. Profile of uploaders

**Figure 3.**
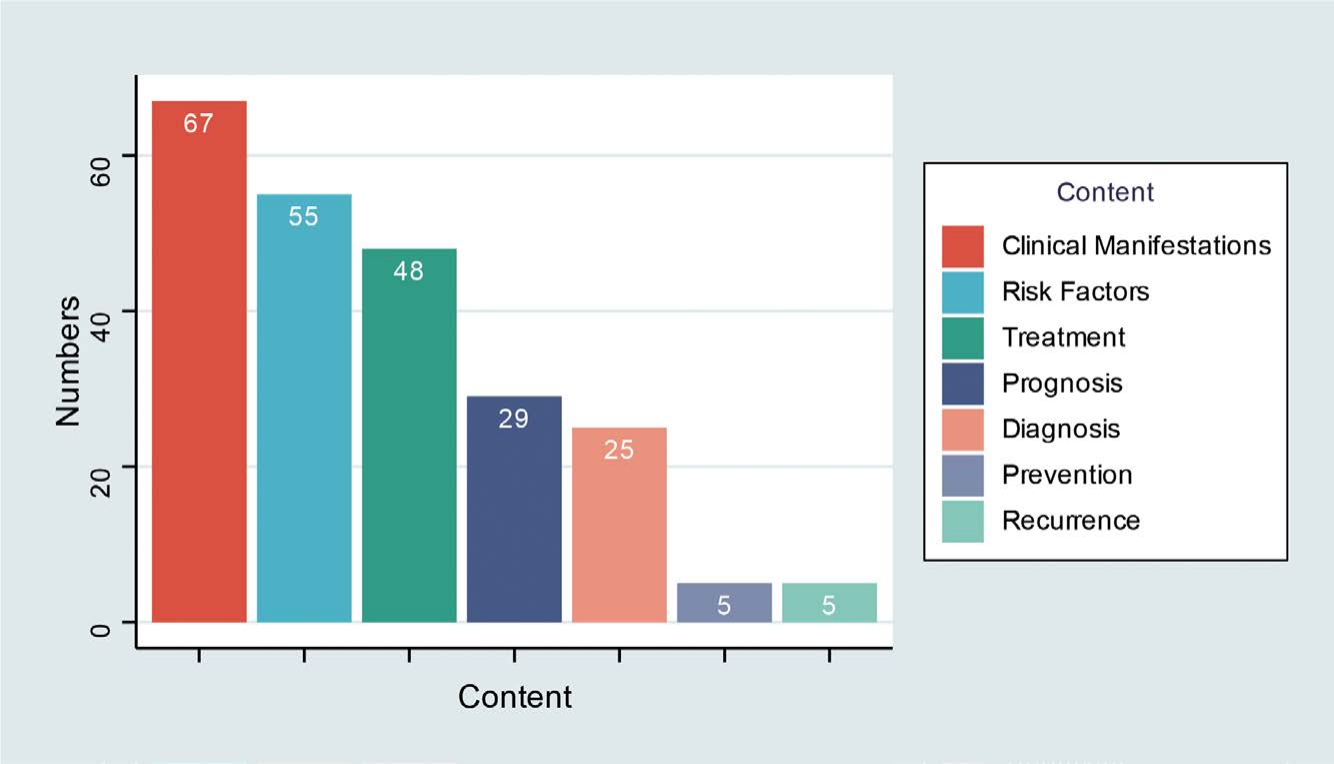
Distribution of thematic content categories across the analyzed videos.

The majority of videos were uploaded by oncologists and dermatologists (Fig. 3), with other uploader categories detailed in Table 4.

**Table 4:** Uploader of short videos.

Uploader	Number (%)
Oncologist	51 (45.13)
Dermatologist	31 (27.43)
Plastic surgeon	11 (9.73)
Orthopedic surgeon	3 (2.65)
Colorectal surgeon	1 (0.09)
Burn surgeon	1 (0.09)
Gastroenterologist	1 (0.09)
Cardiologist	1 (0.09)
Hematologist	1 (0.09)
Critical care physician	1 (0.09)

### 3.4. Comparison of video quality and reliability across different uploaders

**Figure 4.**
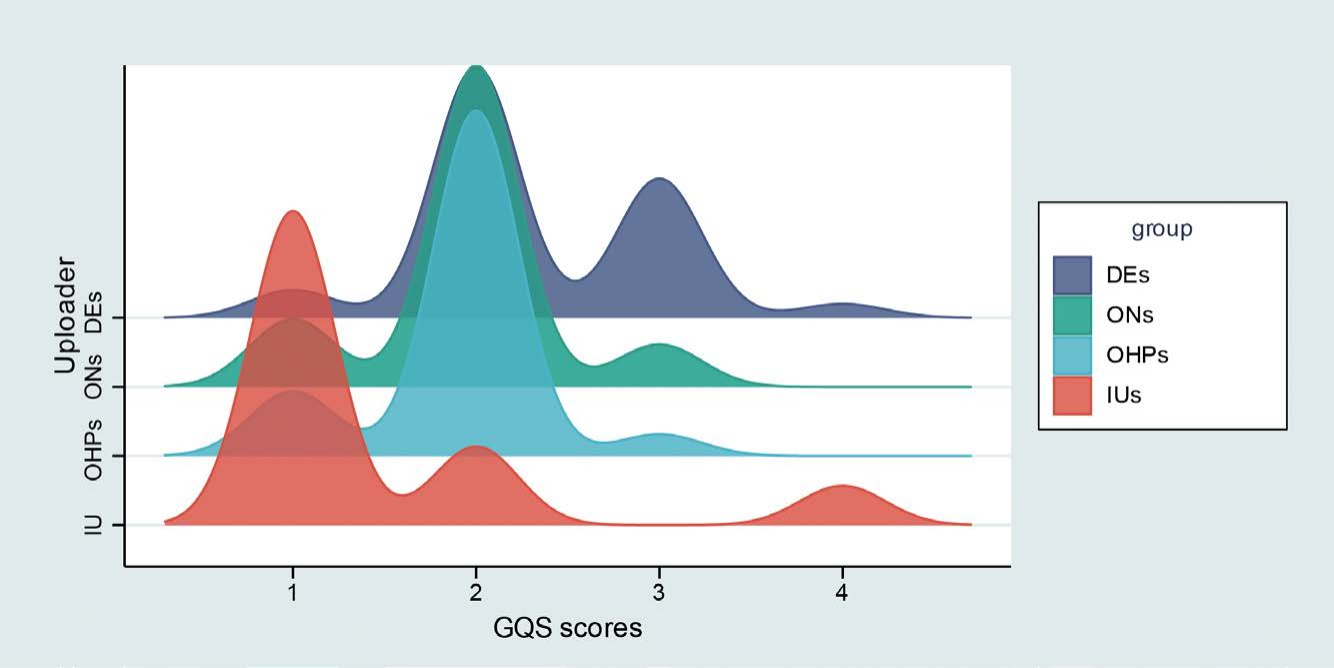
Distribution patterns of GQS scores by uploader category. DEs = dermatologists, GQS = global quality score, IUs = individual users, ONs = oncologists, OHPs = other healthcare professionals.

**Figure 5.**
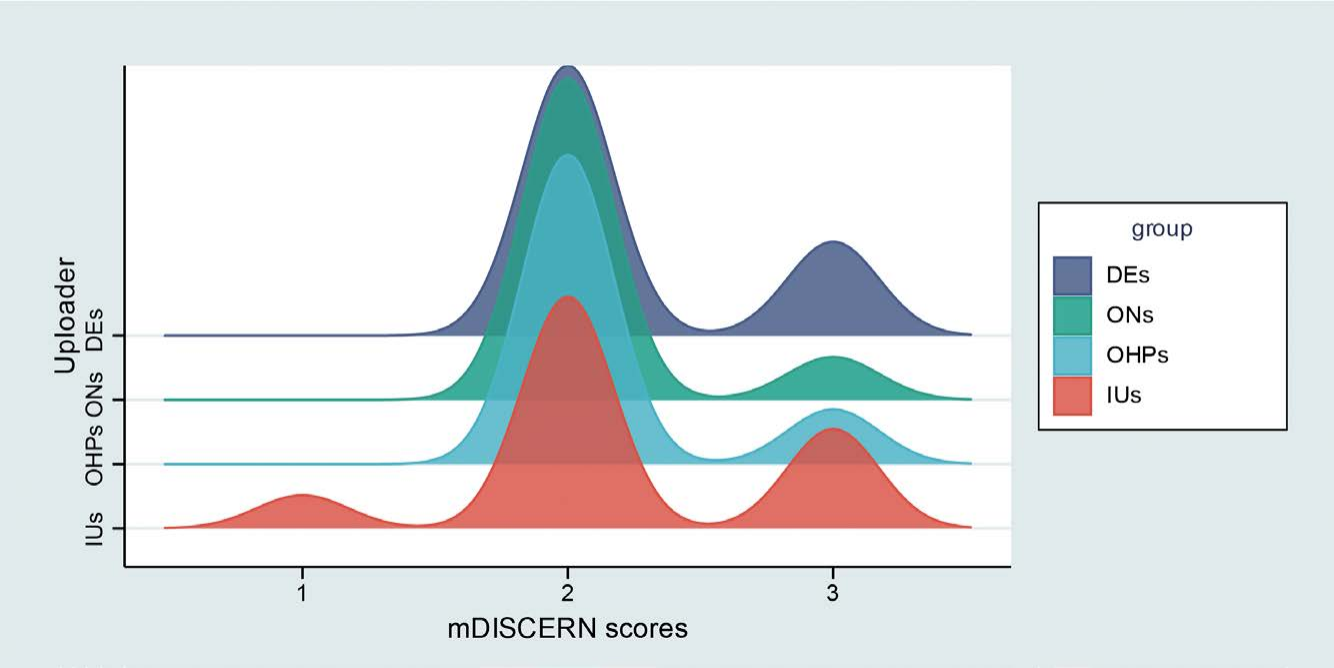
Distribution patterns of mDISCERN scores by uploader category. DEs = dermatologists, IUs = individual users, OHPs = other healthcare professionals, ONs = oncologists.

**Figure 6.**
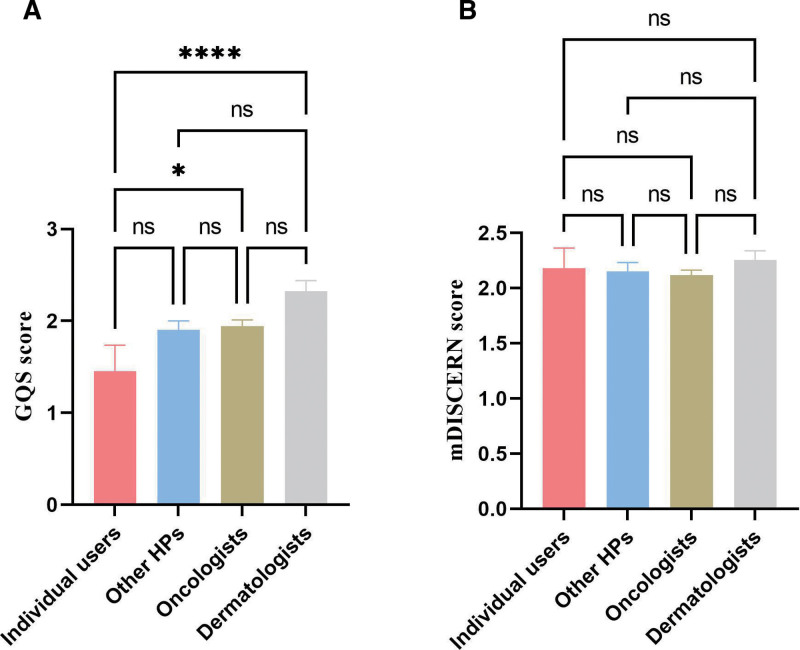
Comparison of GQS and mDISCERN scores across different uploaders. (A) Comparison of GQS across different uploaders. (B) Comparison of mDISCERN scores across different uploaders. * indicates *P* <.05, **** indicates *P* <.0001. GQS = global quality score, ns = not significant.

The distribution of GQS and mDISCERN scores among different uploader types is shown in Figures 4 and 5. Dermatologists had higher GQS scores than oncologists and other healthcare professionals, although the differences were not statistically significant. Both dermatologists and oncologists had significantly higher GQS scores compared with individual users, while other healthcare professionals also scored higher than individual users but without statistical significance (Fig. 6A). No significant differences in mDISCERN scores were observed across uploader types (Fig. 6B). Detailed score parameters are provided in Table 5.

**Table 5:** Differences in video characteristics and quality among dermatologists, oncologists, individual users and other healthcare professionals.

Variables	Dermatologists (n = 31)	Individual users (n = 11)	Oncologists (n = 51)	Other HPs (n = 20)	*P*
Video length, M (Q₁, Q₃)	55.00 (35.50, 71.50)	76.00 (59.50, 246.00)	50.00 (38.00, 67.00)	84.00 (52.75, 127.25)	**.004**
Likes, M (Q₁, Q₃)	1264.00 (128.50, 7359.00)	8302.00 (1373.00, 147552.00)	354.00 (199.50, 948.50)	690.00 (266.50, 5019.75)	**.005**
Collections, M (Q_1_, Q_3_)	395.00 (48.50, 2555.00)	1746.00 (204.50, 6520.00)	93.00 (56.00, 261.00)	237.50 (50.75, 900.25)	**.012**
Comments, M (Q_1_, Q_3)_	194.00 (19.00, 6037.50)	468.00 (60.50, 12894.50)	72.00 (35.00, 448.00)	181.00 (21.25, 1508.25)	.210
Shares, M (Q_1_, Q_3_)	302.00 (61.00, 6746.50)	726.00 (103.00, 29684.00)	68.00 (32.00, 176.50)	444.50 (56.50, 2418.25)	**.004**
GQS scores, M (Q_1_, Q_3_)	2.00 (2.00, 3.00)	1.00 (1.00, 1.50)	2.00 (2.00, 2.00)	2.00 (2.00, 2.00)	**<.001**
Mdiscern scores, M (Q_1_, Q_3_)	2.00 (2.00, 2.50)	2.00 (2.00, 2.50)	2.00 (2.00, 2.00)	2.00 (2.00, 2.00)	.467

Bold values indicate *P* < .05.

M = median, Other HPs = other healthcare professionals, Q₁ = 1st quartile, Q₃ = 3st quartile.

### 3.5. Association between engagement metrics and information quality and reliability

**Figure 7.**
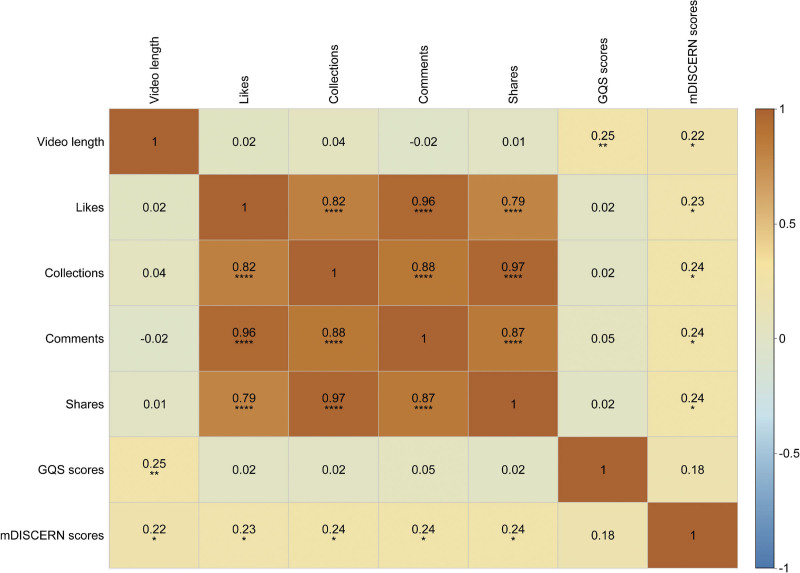
Correlation heatmap of video characteristics, engagement metrics, and quality scores for melanoma-related short videos on TikTok. * indicates *P* <.05, ** indicates *P* <.01, *** indicates *P* <.001, and **** indicates *P* <.0001.

Video duration showed a weak positive correlation with both GQS and mDISCERN scores. Strong positive correlations were observed among engagement metrics, while engagement metrics demonstrated weak positive correlations with mDISCERN scores. No correlation was found between GQS and mDISCERN scores (Fig. 7).

## 4. Discussion

This study evaluated the characteristics, content distribution, quality, and reliability of melanoma-related short videos on the TikTok platform. By analyzing 113 Chinese-language short videos, we found that these videos were generally short in duration and exhibited high levels of user engagement. However, their overall quality and reliability were low. Most videos focused on risk factors, clinical manifestations, and treatment, while content related to prevention and recurrence was notably underrepresented. Further analysis revealed significant differences in content quality among different uploader types, with videos produced by dermatologists achieving the highest-quality scores. These findings have important implications for enhancing the professionalism and comprehensiveness of melanoma-related health communication on digital platforms, as well as for informing strategies for healthcare professionals and platform-level governance.

### 4.1. Video content, quality, and reliability

Content analysis revealed that risk factors, clinical manifestations, and treatment were the most frequently discussed topics, whereas prevention and recurrence were notably underrepresented. This thematic imbalance suggests that current melanoma-related videos on TikTok emphasize disease presentation and therapeutic interventions, while paying insufficient attention to preventive measures and long-term follow-up, which are critical components of public health education. Given that melanoma is the most aggressive type of skin cancer, with high metastatic potential and poor prognosis at advanced stages, the lack of prevention-related information may hinder early detection and delay medical consultation, potentially leading to serious outcomes such as cancer metastasis and death.^[24,25]^ Preventive strategies, including regular skin self-examinations, timely medical visits, and adequate sun protection, are essential for reducing melanoma incidence and improving outcomes.^[26,27]^ The current content gap may represent a missed opportunity for promoting these health behaviors among the public. The overall quality and reliability of the videos were suboptimal, with both GQS and mDISCERN scores showing a median of 2.00 (IQR: 2.00–2.00), indicating that most videos lacked structural completeness, balance, and authoritative references. These findings are consistent with previous research evaluating the quality of disease-related short videos on TikTok and other platforms. One study reported that cervical cancer–related short videos on TikTok were of low quality and reliability, suggesting that patients should exercise caution when interpreting such content.^[28]^ In addition, another multi-platform study found that esophageal cancer–related videos generally had moderate quality and reliability, highlighting the need for improvement.^[29]^ These results underscore the importance for TikTok users to remain cautious when seeking health information online and emphasize the need for healthcare professionals to improve the scientific rigor and accuracy of the content they publish.

### 4.2. Differences in video quality and reliability across uploaders

Significant differences in video quality were observed across uploader types. Videos uploaded by dermatologists achieved the highest GQS scores, followed by those from oncologists, whereas content produced by other healthcare professionals and individual users scored lower. This pattern highlights the importance of the uploader’s professional background in ensuring both medical accuracy and educational value.^[30]^ With their specialized knowledge and clinical experience, dermatologists and oncologists are more likely to provide information that is structurally coherent and strongly evidence-based. Similar patterns have been observed in other health-related studies, indicating that videos uploaded by healthcare professionals generally outperform those produced by nonprofessional users in terms of quality. One study evaluating short videos on pediatric pneumonia across 3 platforms found that, compared with content produced by nonmedical practitioners, videos created by healthcare professionals achieved significantly higher quality assessment scores.^[31]^ Comparable findings have also been reported in research on premature ovarian failure,^[32]^ dry eye disease,^[33]^ and systemic lupus erythematosus.^[34]^ However, even videos uploaded by dermatologists did not consistently meet high-quality standards, suggesting that a medical background alone does not ensure optimal health communication in the digital environment. Training in digital health communication, including concise presentation of health information, targeted audience engagement, and effective visual design, could enhance healthcare professionals’ ability to produce content that is both scientifically rigorous and easily understood by the public.

### 4.3. Correlation between engagement metrics and video quality and reliability

This study observed significant positive correlations among engagement metrics such as likes, comments, shares, and saves, indicating that viewers’ interactive behaviors often occur concurrently. However, no significant correlation was found between engagement metrics and GQS scores. This suggests that high user engagement does not necessarily indicate high-quality educational content. This finding is consistent with previous research in digital health communication where studies on thyroid eye disease and premature ovarian failure also demonstrated no correlation between video engagement metrics and video quality.^[32,35]^ It implies that popular videos may often rely on visual appeal or emotional resonance to achieve high engagement rather than the quality of the information. The TikTok recommendation algorithm tends to prioritize highly engaged content which may inadvertently amplify low-quality or misleading health information. To address this problem, platform-level interventions are needed, including incorporating quality indicators into recommendation algorithms and offering optional quality certification labels for videos that meet established standards. In addition, this study found a weak positive correlation between video duration and GQS scores. This indicates that longer videos may enhance educational value by providing more comprehensive explanations, better-structured narratives, and appropriate references.^[36]^ However, an extended duration alone is not sufficient to guarantee quality. Truly high-quality content must ensure accuracy, relevance, and accessibility to the target audience.

### 4.4. Implications of the findings

This study provides empirical evidence regarding the quality and reliability of melanoma-related health information on the TikTok platform. By applying validated evaluation tools, namely the GQS and mDISCERN, in combination with stratified analyses based on uploader type, we found that the overall quality of video content was generally suboptimal and underscored the important role of professional medical creators in enhancing health communication. The findings also suggest that, under strict content quality standards, moderately extending video duration may help improve educational value. Furthermore, this study identified thematic gaps, particularly in prevention and recurrence, highlighting the need for more comprehensive coverage of melanoma-related content to raise public health awareness and facilitate early detection.

### 4.5. Limitations

This study has several limitations. First, it only analyzed Chinese-language videos on the TikTok platform, which may limit the generalizability of the findings to other languages or cultural contexts. Second, although the sample of 113 videos provided valuable insights, the relatively small sample size may not fully capture the diversity of melanoma-related content on the platform. Third, the assessment of quality and reliability relied on the GQS and mDISCERN scores, and although both tools are widely used, a certain degree of subjectivity is inevitable. Future research could incorporate larger and multilingual datasets, employ more objective quality assessment instruments, and investigate the actual impact of such videos on the public’s knowledge, attitudes, and behaviors regarding melanoma prevention and management.

## 5. Conclusion

This study evaluated the characteristics, content distribution, quality, and reliability of Chinese-language melanoma-related short videos on TikTok. The results showed that although these videos achieved high audience engagement, their overall quality and reliability were low. The content primarily focused on risk factors, clinical manifestations, and treatment, with insufficient coverage of prevention and recurrence. Significant differences in video quality were observed among different uploader types, with dermatologists producing the highest-quality content. Furthermore, no correlation was found between engagement metrics and information quality. This study provides empirical evidence on the current status of melanoma-related health information in the social media environment, offering a valuable reference for optimizing digital health communication strategies, improving the comprehensiveness of health education materials, and establishing platform-level quality control mechanisms.

## Acknowledgments

The authors express their gratitude to the Dermatology Department of the Second Affiliated Hospital of Xi’an Jiaotong University and its leadership for their support.

## Author contributions

**Conceptualization:** Landong Ren, Kaidi Zhao.

**Data curation:** Qinxiao Li, Miao Qin.

**Formal analysis:** Landong Ren.

**Investigation:** Landong Ren, Qinxiao Li, Miao Qin.

**Methodology:** Landong Ren.

**Project administration:** Kaidi Zhao.

**Supervision:** Kaidi Zhao.

**Writing – original draft:** Landong Ren.

**Writing – review & editing:** Kaidi Zhao.
